# Sensory analysis of formulations containing whey protein to individuals undergoing bariatric and metabolic surgery

**DOI:** 10.1186/s12893-023-02004-8

**Published:** 2023-05-12

**Authors:** Tatiana Souza Alvarez, Maria Carolina Batista Campos Von Atzingen, Roseli Oselka Saccardo Sarni

**Affiliations:** 1 Nutrition Department of the Centro Universitário FMABC, Av. Lauro Gomes, 2000 – Sacadura Cabral, Santo André, São Paulo CEP 09060-870 Brazil; 2 Department of Nutrition at the Faculty of Public Health, University of São Paulo, São Paulo, Brazil; 3 Pediatrics Department of the Centro Universitário FMABC, Santo André, Brazil

**Keywords:** Taste, Whey proteins, Bariatric surgery, Gastric bypass

## Abstract

**Background:**

Individuals undergoing bariatric surgery often have inadequate protein intake, which can cause loss of lean body mass, low level of physical activity and sarcopenia. The whey protein supplement is the most suitable in this situation, however there is a low adherence to long-term use due to the palatability and monotony of the recipes. The aim this study was to analyze the acceptability of recipes containing whey-based protein supplements in individuals undergoing bariatric and metabolic surgery.

**Methods:**

An on-demand sampling was performed, through a prospective, experimental study, with individuals undergoing bariatric surgery, treated by a multidisciplinary team, in a clinic located in São Paulo, Brazil. The study excluded: individuals with possible changes in taste during the sensory testing period. The study was divided into selection of recipes containing whey proteins, recruitment of tasters, sensory and chemical analysis of the recipes.

**Results:**

The sample consisted of 40 tasters, adults, and elderly, who underwent bariatric and metabolic surgery, with a median of eight years of surgery, who had previously consumed a supplement. These individuals were subjected to sensory analysis of six recipes with fresh and minimally processed foods, plus protein supplement. All recipes had food acceptance above 78% and the chemical analysis of the recipes showed an average of 13 g of protein per serving.

**Conclusion:**

There was favorable acceptance of recipes with whey proteins, which places them as good dietary alternatives for the prevention of sarcopenia and weight relapse in individuals undergoing bariatric and metabolic surgery.

## Introduction

A bariatric and metabolic surgery has proved to be effective for the control and treatment of severe obesity [1, 2]. In these individuals, insufficient and irregular proteinintake is common, aside from the low adherence to regular supplement use, which may result in complications, such as sarcopenia, muscle mass loss and level of physical activity [3–5]. In addition to that, the inadequate protein intake, and the return to the pre-surgery dietary pattern with carbohydrates and fat-rich diets, can reduce weight loss or even trigger weight regain [6].

Nutritional post-bariatric guidelines and recommendations suggest the adoption of a relatively high protein intake specially in the first months after surgery. Protein intake should be individualized, assessed, and guided by a registered dietitian. A minimal protein intake of 60 g/day and up to 1.5 g/kg ideal body weight per day should be adequate [1, 2]. For supplementation is indicated to use high biological value proteins.

The muscular protein turnover is influenced by the type of protein and amino acids that are consumed. Essential amino acids cannot be synthesized de novo by the body and must be introduced through the diet. The whey protein is prescribed for its better digestibility, due to high quantities of branched chains amino acids, in particular the leucine, which plays an important role in muscle protein synthesis [7].

Protein intake plays an important role in the regulation of body weight. A protein-rich diet promotes early satiety, facilitates a reduction in total energy intake, increases food-induced thermogenesis, associated with exercise, preserving lean mass. Protein supplementation can contribute to weight loss and prevent the loss of lean mass [7–9].

In the area of sensory testing with whey proteins, there are predominantly publications related to the development of products for the industry and food supplements for oncology patients. According to our knowledge, there are no related studies, if considered the sensorial analysis of formulations containing protein supplementation for individuals submitted to bariatric and metabolic surgery.

The purpose of this study was to analyze the acceptance of formulations containing whey protein in individuals submitted to bariatric and metabolic surgery.

## Methods

### Population profiling

Through a prospective and experimental study, individuals who underwent bariatric and metabolic surgery by videolaparoscopy for more than one year, using Roux-en-Y gastric bypass (RYGB) or sleeve gastrectomy (GV) techniques, were included. In this study, a convenience sampling was performed. The individuals were selected in the nutrition service of a multiprofessional clinic, located in the city of São Paulo, Brazil. Excluded from the study were individuals: aged less than 18 years, who had other surgical procedures; possible changes in taste during the sensory testing period, such as pregnant women, smokers, with severe or acute infectious and/or inflammatory processes, and those who did not complete the sensory test.

The individuals were informed about the research procedures and signed an informed consent form.

The research was approved by the Research Ethics Committee of the FMABC University Center, with number 1.781.449.

**Fig. 1:**
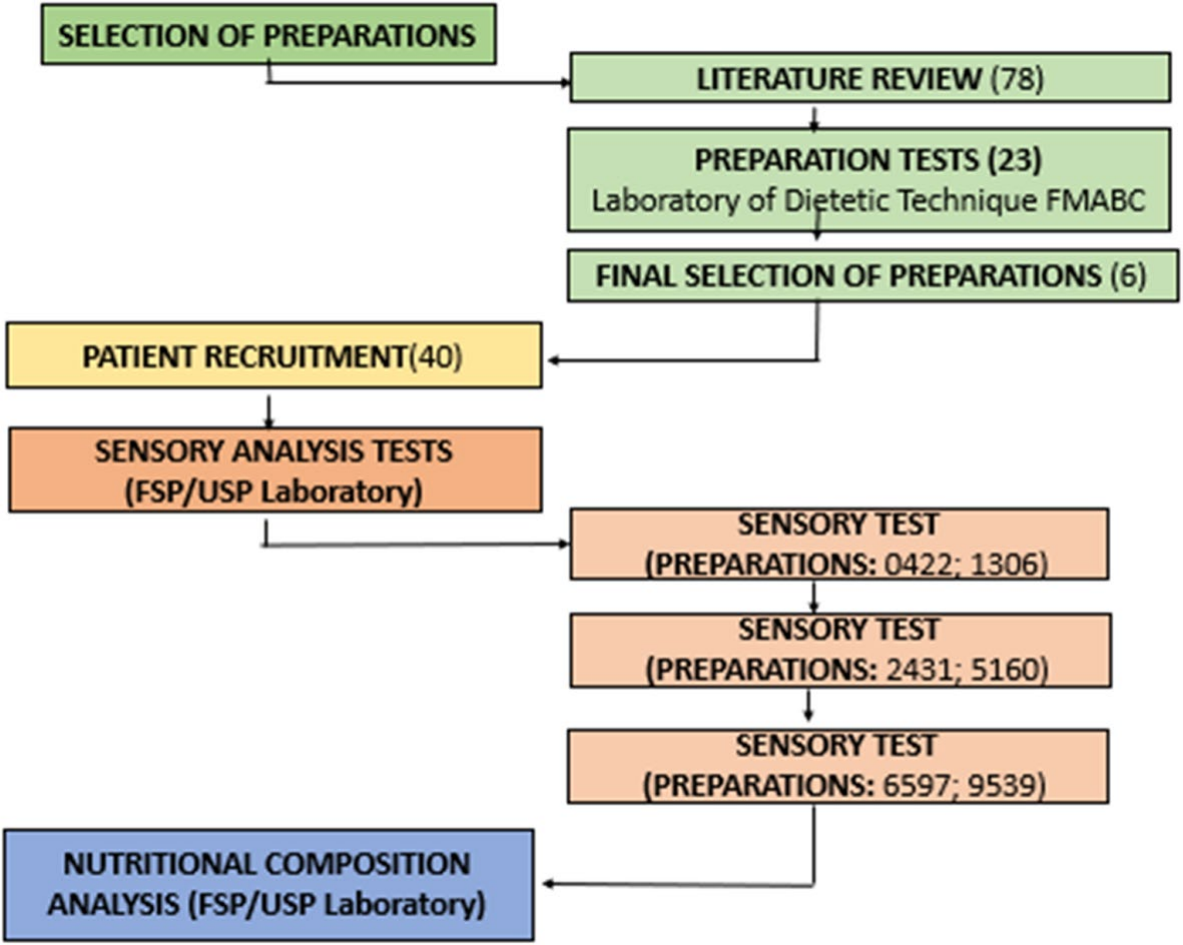
Study flowchart

The study was divided into selection of formulations containing whey proteins, recruitment of panelist, sensory and chemical analysis of the formulations, as described in Fig. 1.

The group of panelists recruited answered a self-completed questionnaire about habits and attitudes towards the consumption of protein supplements, a questionnaire of food recall of a usual day and participated in the hedonic tests of preparations added with protein supplements, for the attributes appearance, flavor, aroma, color and texture.

### Recipes selection

For this work, the authors of this research carried out a bibliographic survey of recipes, with the objective of bringing new possibilities to individuals undergoing bariatric surgery. In the selection of recipes, easy-to-reproduce preparations were considered, containing easily accessible ingredients, accessible cost and foods containing a high protein content. They were tested at the Laboratory of Dietetic Techniques at the Centro Universitário FMABC, Brazil.

Among the 78 selected initial recipes, 23 were separated for the lab tests. Seventeen of those were excluded due to large amounts of ingredients, ultra-processed foods, and the difficulty level in reproducibility. Therefore, six of them were selected to sensorial analysis.

During the selection of the recipes, the following aspects were considered: low sodium and fat content, high protein level and few ingredients, in natura and minimally processed food, easy execution, yield in small portions, sweet and salty to used for consumption throughout the day (snacks and main meals), adapted according to the individuals who were submitted to bariatric and metabolic surgery needs. The ingredients used followed the recommendations of the Food Guide for the Brazilian Population [10].

The selected recipes were chickpea quiche (code 0422), humus (code 1306), meatballs in tomato sauce (code 2431), cocoa bean cookie (code 5160), yam dumpling with meat (code 6597), chocolate flan (code 9539), with the addition of the supplement in all of them.

The supplement in question contained isolated and partially hydrolyzed whey proteins (92% WHI), neutral flavor, brand Vitafor®, purchased by the researcher himself. The supplement used is a source of BCAA, L-glutamine, L-proline and L-arginine, in addition to containing protein peptides such as glycomacropeptides, alpha-lactoalbumin, lactoferrin and low levels of carbohydrates, lipids and sodium.

The dilution of the protein supplement was carried out as follows: the protein supplement was first mixed with the liquids at room temperature of each recipe, to form a paste and homogenized for about 2 to 3 min, then the other ingredients were added following the instructions of each recipe.

### Sensorial analysis

The sensorial analysis was taken place in the Laboratory and Didactic Kitchen of Procedures and Culinary Techniques Applied to Nutrition, School of Public Health, University of São Paulo, Brazil (FSP/USP).

The recipes, after ready, were arranged in identical containers, numbered, and then subjected to a blind experimental study, in which individuals volunteered themselves to taste the food, without knowing the composition of the recipe.

The panelist received the samples in white dishes, which were randomized and coded with three random digits. During the sensory test a small amount of water was offered for the mouthwash, napkins, tableware, and plastic cup. Every two tastings were held a break, of 15 min, considering the gastric volume reduction of the panelists. The volunteer panelists performed the sensory analyses in separated locations, under white light, in the period from August to September 2018.

The acceptance of the formulations was evaluated by means of a seven-point structured hedonic verbal scale, which varied from "very much disliked" (1) to "very much liked" (7), with the five points being equivalent to "moderately liked" [11].

### Determination of the protein content of recipes

The protein was determined using the micro Kjedahl method, conversion factor 6.25 for protein conversion (general factor) and factor 6.38 for the samples whose protein source is exclusively milky, according to the Instituto Adolfo Lutz (1985) [12] pattern, in the Laboratory of Food Components and Health of the School of Public Health of the University of São Paulo, Brazil (FSP/USP).

According to the chemical analysis, the protein supplement in a measured spoon contains 30 g, which are equivalent to 26.51 g of protein.

In recipes 0422, 1306, 2431 and 9539, the protein supplement were 34 g in 100 g of the recipe. In preparations 5160 and 6597, the supplement were 17 g added.

After the chemical analysis of the preparations, the protein amount per portion and in 100 g of recipe was calculated.

### Statistical analysis

The sample calculation was 40 panelists. In order to calculate the sample size, the following parameters were considered: prevalence data of food acceptance of protein supplement in patients who underwent bariatric and metabolic surgery of 20%, confidence adopted level of 95%, sample error of five percentual points, and 80% testing power for two-tailed testing [13].

For the data analysis was used a statistical program Stata version 14.0. The qualitative variables were presented in tables by absolute frequency and relative frequency. To compare gender and type of surgery, according to sensory tests, Fisher’s exact and chi-square tests were used. The significance level was 95% and *p* < 0.05 was considered significant.

## Results

The sample was composed of 40 individuals who underwent bariatric and metabolic surgery, technics of Roux-en-Y Gastric Bypass (*n* = 25) and Vertical Gastrectomy (*n* = 15), being 27.5% of the male gender (*n* = 11) and 72.5% of the female gender, ages between 18 and 77 years old, as describes in Table 1. The average time of surgery was eight years, ranging from 3 to 17 years.

**Table 1:** Characteristics of the studied sample (*n* = 40)

Variable		N	%
**Type of surgery**	Roux-en-Y Gastric Bypass	25	62.5
	Vertical Gastrectomy	15	37.5
**Gender**	Male	11	27.5
	Female	29	72.5
**Age range (years)**	18 – 35	5	12.5
	36 – 59	28	70.0
	Above 60	7	17.5
**Marital Status**	Single	28	70.0
	Married	9	22.5
	Divorced	3	7.5
**Race**	Caucasoid	29	72.5
	Non-caucasoid	11	27.5
**Education**	Elementary School	0	0
	High School	13	32.5
	College	27	67.5
**Occupation**	Retired	4	10.0
	Health Professional	5	12.5
	Administrative	31	77.5
**Surgery time (years)**	3 – 5	10	25.0
	6 – 10	19	47.5
	> 10	14	35.0
**Consumption of protein supplement (currently)**	Yes	12	30.0
	No	28	70.0
**Start of protein consumption**	First week of surgery	13	32.5
	First 15 days after surgery	11	27.5
	First month of surgery	14	35.0
	Do not you remember	2	5.0
**Duration of protein intake**	Three months	16	40.0
	Six months	16	40.0
	One year	8	20.0
**Consumption of protein supplement (currently)**	Yes	12	30.0
	No	28	70.0
**Importance of protein supplement consumption**	Lean mass maintenance	30	75.0
	Better nutrient absorption	5	12.5
	Aid in weight loss	5	12.5

All individuals undergoing bariatric surgery were instructed to consume a protein supplement soon after the procedure, in accordance with the guidelines [1, 2]. The data described in Table 1, refer to the protein consumption history of these individuals.

The beginning and duration of supplement use varied among participants, due to gastrointestinal discomfort, possibly caused by dietary errors, such as: aerophagia, nausea, vomiting, insufficient chewing. In these cases, they were reoriented on appropriate eating behaviors and encouragement to increase the consumption of protein-source foods and industrialized products added with protein. Regarding the difficulty in consuming the protein supplement, 52% individuals (*n* = 21) reported low adherence to supplementation due to taste, texture alteration in recipe, cost, and gastrointestinal discomfort.

Regarding the individuals who reported the consumption of protein supplementation routinely (*n* = 12), described in Table 1, when analyzing the usual food recall, it was observed that the protein consumption of these individuals was 82.7 ± 30.9 g per day, while the group that did not usually consume protein supplementation was 77.7 ± 18.3 g per day, however there was no statistically significant difference between the groups.

Regarding the selected recipes, ingredients with high protein content, in natura and minimally processed foods stand out, according to the guideline of the Food Guide for the Brazilian Population [10], described in Table 2.

**Table 2:** List of the main ingredients with the respective quantities used in the recipes

**Code** ^a^	**Ingredients**	**Quantity (g/ml)**	**Yield (portions)**
**0422**	Cooked chickpeas	403	5
	Oatmeal flour	30	
	Canola oil	17	
	Whey protein isolated	34	
	Egg	55	
	Skimmed milk	25	
	Ricotta	50.7	
	Leek	19.5	
**1306**	Cooked chickpeas	294	4
	Tahini	31	
	Lime	51	
	Garlic	7	
	Whey protein	34	
**2431**	Lean ground beef	100	7
	Onion	163	
	Tomato	170	
	Garlic	13	
	Olive oil	20	
	Whey protein	34	
**5160**	Black beans	290.5	3
	White sesame	15	
	Cocoa powder	14,5	
	Tahini	16.5	
	Whey protein	17	
**6597**	Yam	104	3
	Broccoli	44	
	Egg	55	
	Lean ground beef	87	
	Whey protein	17	
**9539**	Cocoa powder	15	3
	Cornstarch	9	
	Egg	55	
	Skimmed milk	231	
	Whey protein isolated	34	

^a^Recipes: 0422—chickpeas and ricotta quiche; 1306—humus; 2431—meatballs in tomato sauce; 5160—bean cookie with cocoa; 6597—yam dumpling with meat; 9539—chocolate flan

In the description of the ingredients, aromatic seasonings, salt and sweetener were excluded because they did not have an energy value (Table 2). The protein content analysis is presented in Table 3, per serving and per 100 g of preparation.

**Table 3:** Protein’s medium values and standard deviations

Code	Yield (homemade measure/g)	Added protein supplement	Protein (g)	
			**Portion**	**100 g**
**0422**	3 units (75 g)	**Yes**	9.71 ± 0.81	17.19 ± 1.02
		**No**	6.13	8.18
**1306**	3 tablespoons (87 g)	**Yes**	6.91 ± 0.33	13.81 ± 0.67
		**No**	4.18	4.80
**2431**	3 units (76 g)	**Yes**	16.50 ± 0.47	22.14 ± 0.63
		**No**	9.90	13.13
**5160**	2 units (80 g)	**Yes**	10.76 ± 0.64	16.28 ± 0.97
		**No**	9.42	11.78
**6597**	4 units (87 g)	**Yes**	17.17 ± 0.86	21.51 ± 1.07
		**No**	14.80	17.01
**9539**	4 tablespoons (110 g)	**Yes**	15.22 ± 0.10	18.27 ± 0.12
		**No**	10.19	9.26

The average amount of protein in the recipes without the addition of a protein supplement was 10.70 g in 100 g of food, while when supplements were added to the recipes, they now contained 18.20 g of protein in 100 g of the recipe.

From the results obtained from the sensory analysis, a value of five was considered as acceptable, and values ​​lower than a grade of five as not acceptable. All recipes were accepted by the panelists, with no statistically significant difference between formulations.

**Fig. 2:**
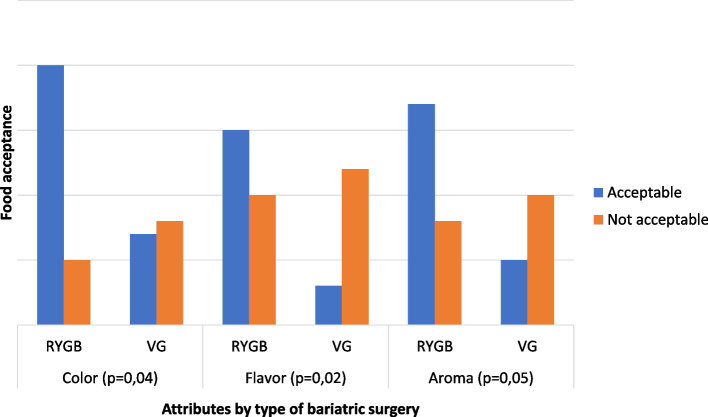
Acceptance of the attributes color, flavor, and aroma in recipe 6597 by type of bariatric surgery

Regarding the surgical technique, stands out the recipe 6597 in the attributes color, flavor and aroma, there was a significant difference between the panelists (Fig. 2).

**Fig. 3:**
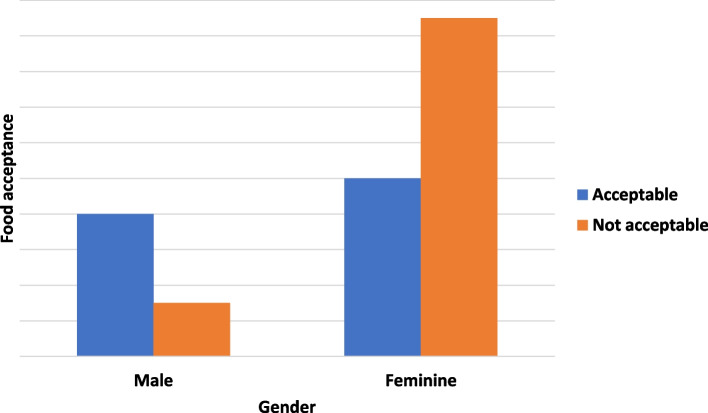
Flavor attribute acceptance by gender in recipe 9539

Comparing the analysis by gender, a statistically significant difference was found in recipe 9539 regarding taste (Fig. 3). The male gender reported good acceptance of recipe 9539 when compared to the female gender.

After the sensory analysis of the formulations, the individuals received the recipes and were asked about the intention to prepare, 71% reported that these formulations (*n* = 29) could be reproduced in their homes usually.

## Discussion

The present study carried out with individuals submitted, for an average time of 8 years, to bariatric and metabolic surgery, showed that all the offered formulations, containing whey protein supplement had satisfactory and higher acceptance than the average value. All the formulations used had a high protein value, prioritizing the use of fresh and minimally processed foods (snacks and meals). The sensorial analysis revealed the acceptance over 78% of all formulations.

Individuals who underwent the surgical procedure with a longer duration of surgery were selected so that there was no interference in the acceptability of the recipes, since in the first year of surgery there are many changes in taste and adaptation of the consistency of the diet.

In the selection of recipes, the food groups of legumes, meat and milk and dairy products prevailed so that the daily protein targets can be achieved.

Despite the absence of other similar experimental studies for comparison, these findings are in line with other publications that show the importance of strategies to reach the daily protein goal, through a high-protein diet, supplementation, as well as the elaboration of recipes adapted to individuals submitted to bariatric surgery proposed in this work [14, 15].

Whey protein supplementation after bariatric surgery has a beneficial effect enhancing the loss of body fat tissue and reducing the loss of lean body mass [7]. It is common that the surgery leads to the individual’s difficulty in consuming foods rich in fibrous protein foods, such as beef and fish. To meet the high protein needs, it is suggested to increase the intake of other less fibrous dietary sources with high biological value and to perform different cooking processes [2, 14].

According to Cambi et al., the whey protein supplement intake should be encouraged in liquid formulations, as observed in clinical practice, however through this study it was possible to test different ways of using this supplement in recipes for greater adherence in the long term. [16, 17]

Regarding the difficulty in consuming the protein supplement in the late postoperative period, to achieve protein goals, several authors mention the change in taste and texture after adding the supplement in the formulation, as well as some reports of intestinal discomfort and high cost, as also observed. in this study [7–9, 18].

Based on the results of the chemical analysis, the recipes presented in this study contained an average of 12.71 g of protein per serving. Considering the recommendations to prevent sarcopenia, the intake of 25 to 30 g of protein per meal, distributed in three main meals, a portion of the formulations presented in this paper already contributes approximately 51% of the lower recommended value. When considering the recommended guidelines for bariatric and metabolic surgery, a minimum of five meals and 60 g of protein per day, in one meal the ideal would be the consumption of at least 12 g of protein, therefore a portion of these formulations provides an approximate contribution of 100% of the recommendations [1, 2, 19].

Faria et al., refer that when selecting recipes for individuals undergoing bariatric surgery, a minimum amount of protein per recipe of 10 g for snacks and 20 g for main meals should be considered. Therefore, all recipes tested in this study exceed the guidance of these authors [20].

The tested recipes were prepared as part of the main meals of individuals undergoing bariatric surgery, when analyzing recipes 0422 and 2431 added with supplementation, they contain 9.71 and 16.50 g of protein, respectively, representing 65% of the protein content of a meal for this public. If these recipes were consumed without supplementation, they represent 40% of the protein recommendation in a daily meal.

The other recipes (1306, 5160, 6597 and 9539) can be part of these individuals' snacks. These recipes with or without supplementation meet the recommendation of a minimum of 10 g of protein per serving. The only exception is recipe 1306, which must be consumed with another protein source food to complement the recommendation for that meal.

In the sensorial analysis of recipes 6597 and 9539, the attributes color and flavor showed significant difference when separated by type of bariatric surgery and gender.

The data from the hedonic scale of formulation 6597 according to color, flavor and aroma criteria, and the type of surgery showed a preference in the acceptance of individuals undergoing the Roux-en-Y Gastric Bypass technique, when compared to those undergoing Vertical Gastrectomy. Literature data confirm that the anatomical and metabolic changes associated with RYGB show greater acceptance of salty formulations, due to a tendency to decrease the values of perception of sweet taste [21–23].

Smith et al. (2021) suggest that bariatric surgery highlights neurobiological reward mechanisms in taste preference, which may correct the brain responses to food stimuli, high in sucrose and fat in the circuits that mediate reward function. Those changes in taste preference may affect the food intake and weight reduction [22].

The systematic review by Alhanouf and colleagues (2022) provided evidence confirming postoperative changes in sensitivity to four taste domains after different bariatric procedures. Patients with Vertical Gastrectomy showed increased sensitivity for four taste domains (sweet, sour, salty, and bitter). However, patients with RYGB showed a variable pattern in taste perception, changes included a short-term increase in detection and a decrease in sweet taste preference, in addition to increased sensitivity to salty and sour taste, confirming literature data [23].

Both surgical procedures, RYGB and VG, promote modifications in the gastrointestinal tract, that result in dysbiosis, promoting differences in the composition of the salivary and fecal microbiota and bacteria-derived metabolites such as butyric acid and butyrate. Differences in the microbiota composition profile, including salivary microbiota between individuals with obesity and eutrophic are well documented; however, how these changes affect taste is not fully established [23, 24].

In the present study, 22 individuals rejected the taste of recipe 9539, and when separated by gender, different results were observed. The rejection of the sweet taste was considered by the average of the sensory analysis as less than five. The sweetener presents in this formulation associated with the supplement containing partially hydrolyzed proteins promotes a bitter taste [9, 25]. In males, there was an acceptance of 72.7%, while in females, 34.5%. In the males there was a preference for a sweet taste, and perhaps the presence of certain ingredients in the formulation may have influenced it, such as milk and cocoa. These ingredients contain tryptophan, its metabolites, such as serotonin, play a role in the metabolism and appetite observed in obesity [7], confirming data from several studies on taste modifications after surgery [19–24].

Several studies have shown that most amino acids have flavor. Delompré et al., state that amino acids can contribute to the aftertaste, L-glutamine and L-proline have a sweet taste, L-leucine and L-arginine have a bitter taste [25]. In the recipes containing protein supplement offered to individuals undergoing bariatric surgery, these four amino acids were contained. Therefore, the results of the sensory analysis may have been influenced by the presence of the amino acids contained in the whey protein supplement. To better understand this issue further studies with hyperproteic formulations without supplementation should be conducted.

## Conclusions

The results referring to the attributes evaluated in the sensory analysis showed that the scores were higher than the average value of the scale used, indicating that the selected formulations were well accepted by the panelists. These data show that the action of whey protein on the sensory characteristics of the formulations was positive in individuals undergoing bariatric surgery.

A high-protein diet is extremely important in the postoperative period of bariatric surgery, so dietary strategies should be encouraged, which can contribute to the long-term maintenance of lean mass, prevention of sarcopenia, level of physical activity and weight regain.

Among the limitations of the study, it should be noted that the sensory analysis can be performed in individuals undergoing bariatric surgery in different postoperative periods, to assess the interference of the period and the type of procedure in food acceptance. In addition to the inclusion of liquid recipes, such as soups, juices, ice cream and recipes with sweeteners that do not change their taste when adding protein supplementation.

Further studies with sensory and chemical analysis of formulations containing protein supplements are suggested, with a larger sample size, with different characteristics of protein supplement, including all phases of food consistency after bariatric surgery.

## Data Availability

The dataset of this research is available in repositories available at FMABC University Center. Follow the contact of the main researcher to access the materials – tatiana.alvarez@fmabc.net.
